# Ependymoblastoma with pulmonary metastasis in an adolescent: A case report

**DOI:** 10.3389/fneur.2022.964856

**Published:** 2022-08-09

**Authors:** Xinmin Xu, Angcheng Li, Xia Xu, Qiangjun Gong, Shengjie Zhu, Wenya Chu, Shubo Ding

**Affiliations:** Department of Radiotherapy, Jinhua Municipal Central Hospital, Jinhua, China

**Keywords:** ependymoblastoma, case report, adolescent, pulmonary, metastasis

## Abstract

Ependymoblastoma is a rare embryonal neoplasm of the nervous system, and the entity is even rare with distant metastasis. This case can help refine the existing literature and provide lessons for the management of other patients with ependymoblastoma. The present case concerns an adolescent with supratentorial ependymoblastoma, who received gross-total resection (GTR), postoperative radiotherapy, and six cycles of chemotherapy, with disease-free survival (DFS) of about 5.3 years. Subsequently, pulmonary metastasis occurred, but no intracranial lesion was found. Finally, combined treatment with radiotherapy and chemotherapy significantly reduced the lung lesions, with progression-free survival (PFS) of 10 months and long-term survival of 6.3 years. This case indicates that the lung metastases of ependymoblastoma are relatively sensitive to radiation, but lung metastases have not completely disappeared. Perhaps, increasing the radiation dose to lung metastases can improve the efficacy, which is worth exploring.

## Introduction

Ependymoblastoma is an embryonal tumor of the neuroepithelium, which usually occurs in women and children and is concentrated in the upper part of the tentorium. Ependymoblastoma is rare, highly malignant, and prone to local infiltration and pia meningeal spread (1). Ependymoblastoma is a primitive neuroectodermal tumor (PNET) according to the 2007 central nervous system (CNS) Tumor Classification Guidelines (2). Supratentorial PNETs represent <1% of primary CNS tumors (3). We searched the current database and found no reports of ependymoblastoma lung metastases. Ependymoblastomas are large tumors with well-defined tumor margins; moderate to low signal on T1WI and moderate to high signal on T2WI, with limited diffusion (4). Ependioblastoma has a multilayer rosette structure (1). Immunohistochemistry was positive for vimentin, s-100 protein, EMA, cytokeratin, synaptophysin, and CD99 (1, 5, 6). This case can help to complete the existing literature on ependymoblastoma.

## Case presentation

**Figure 1:**
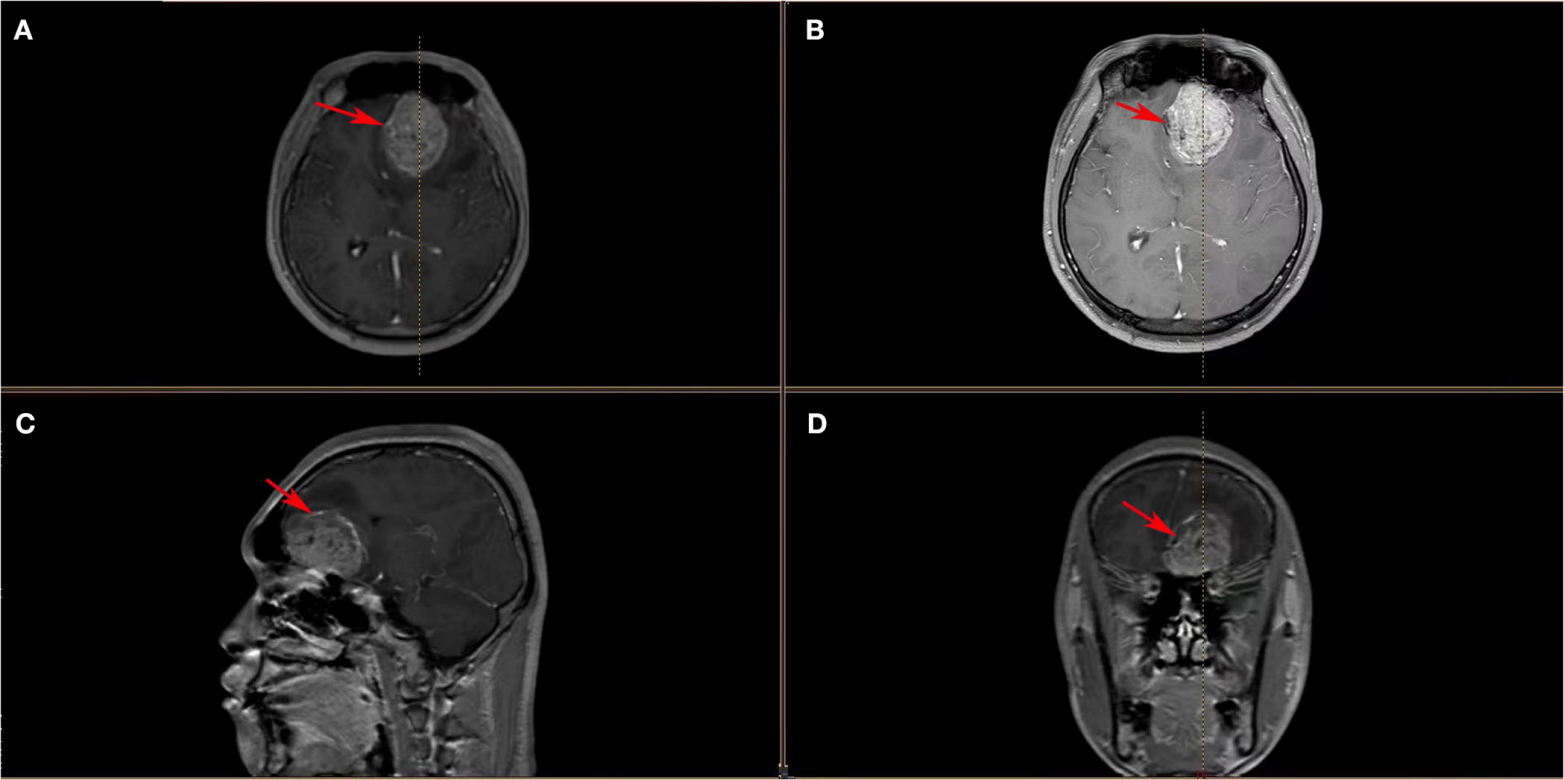
**(A–D)** Head MRI images showed a space-occupying lesion in the left frontal lobe with heterogeneous enhancement of the mass, surrounding edema, right-lateral midline structure, compression narrowing of the lateral ventricle, and shallowing local sulci and cistern (these red arrows indicate ependymoblastoma).

**Figure 2:**
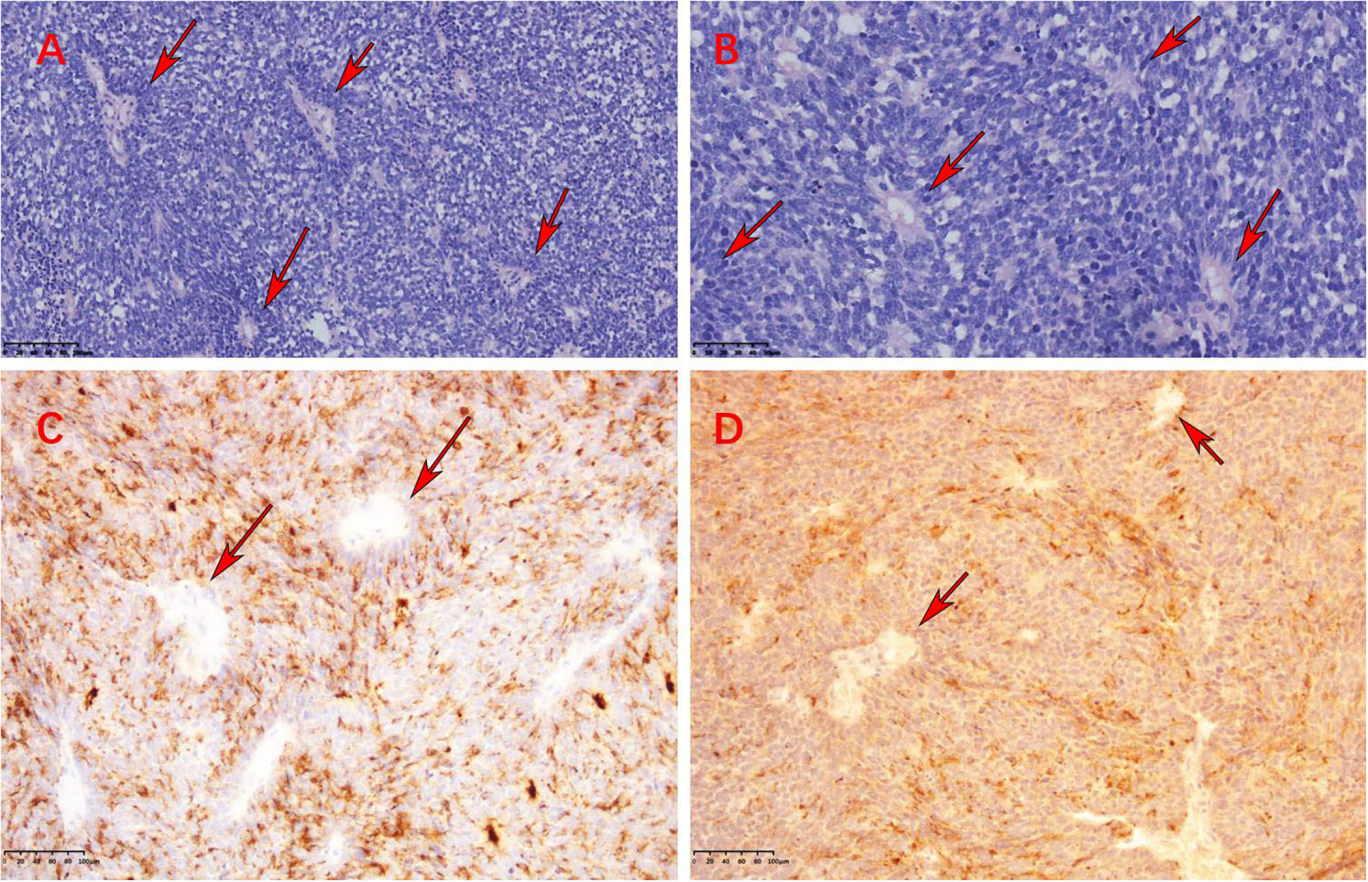
**(A,B)** High magnification view showed multiple rosettes of ependymoblastoma consisting of a concentric arrangement of small blue round cells around the cavity with nuclear pleomorphism and mitotic activity (HE, ×200 and HE, ×400). **(C,D)** Immunohistochemical staining was positive for EMA and Syn, respectively (×100) (these red arrows indicate multiple rosettes).

An 18-year-old male patient presented with a sudden headache with persistent dull pain, primarily in the left forehead, which was unbearable without inducement in 2015. The patient had a healthy lifestyle and had no familiarity with cancers. Neurological examination showed the following: clear consciousness, normal pupil light reflex, normal muscle tone, muscle strength level 5, cranial nerve physical examination negative, and bilateral pathological reflex sign negative (no positive signs). Head MRI suggested that the lesion was in the left frontal lobe, about 4.8 × 4.3 cm in size, and T1WI showed uneven enhancement of the lesion (Figure 1). Preoperative laboratory examination showed no abnormalities. In December 2015, the patient underwent craniotomy for tumor resection under general anesthesia. The patient was pathologically diagnosed with a PNET in our hospital, which was confirmed as left frontal ventricle ependymoblastoma by pathologic consultation in a superior hospital (Figures 2A,B), with a multi-layer rosette structure. The immunohistochemical staining was positive for EMA (Figure 2C) and Syn (Figure 2D) expressions.

**Figure 3:**
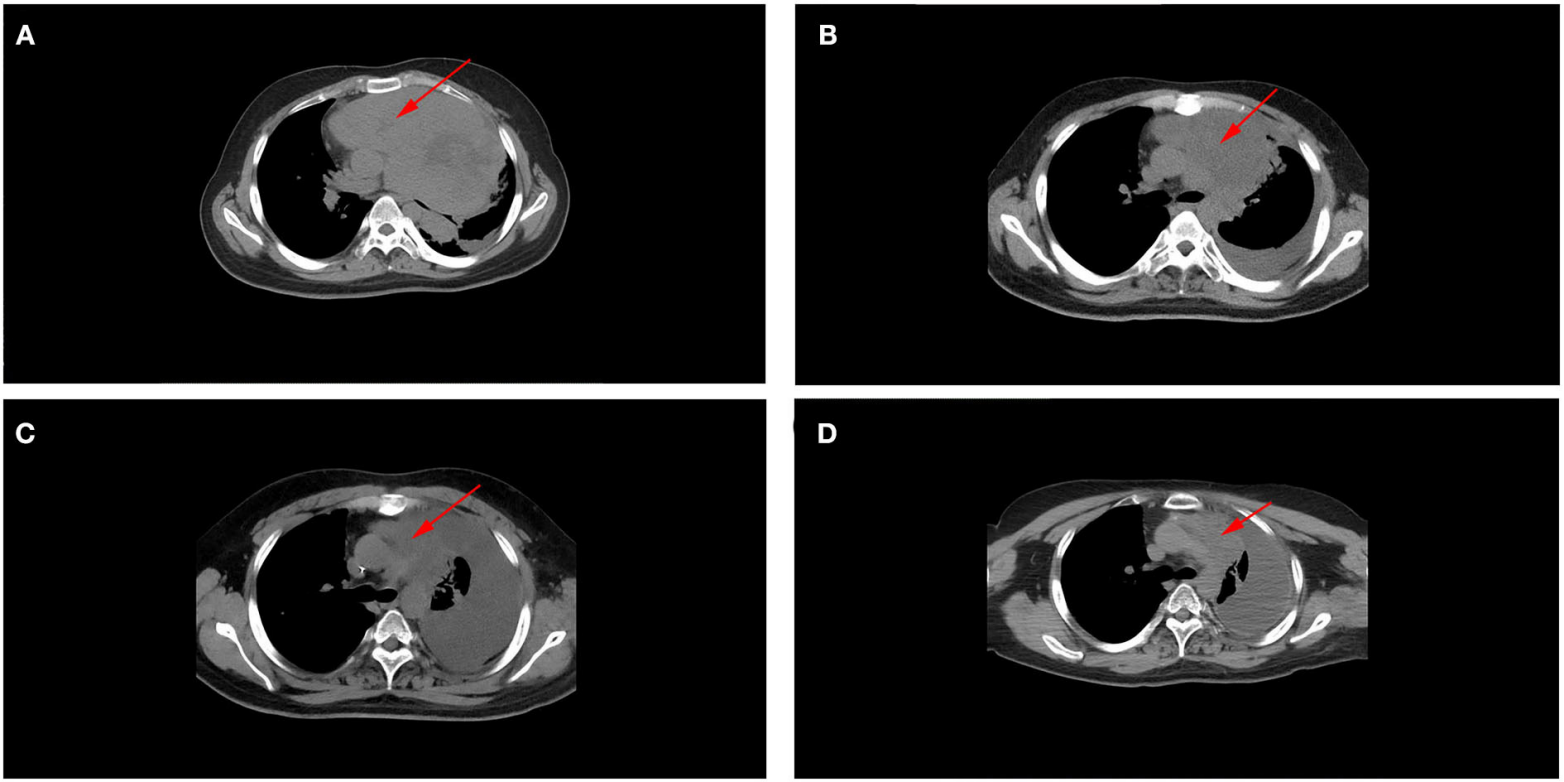
**(A)** Chest CT scan (before treatment, after radiotherapy, after chemotherapy, and at a recent point), showed a large space-occupying lesion with bronchial occlusion in the left thoracic cavity with an unclear mediastinum boundary. **(B)** As treatment continued, the lesions gradually shrank, and left pleural effusion increased. **(C,D)** No significant change was observed in lung metastases between the end of chemotherapy and the last follow-up period, and they were similar in size (these red arrows indicate lung metastases).

**Figure 4:**
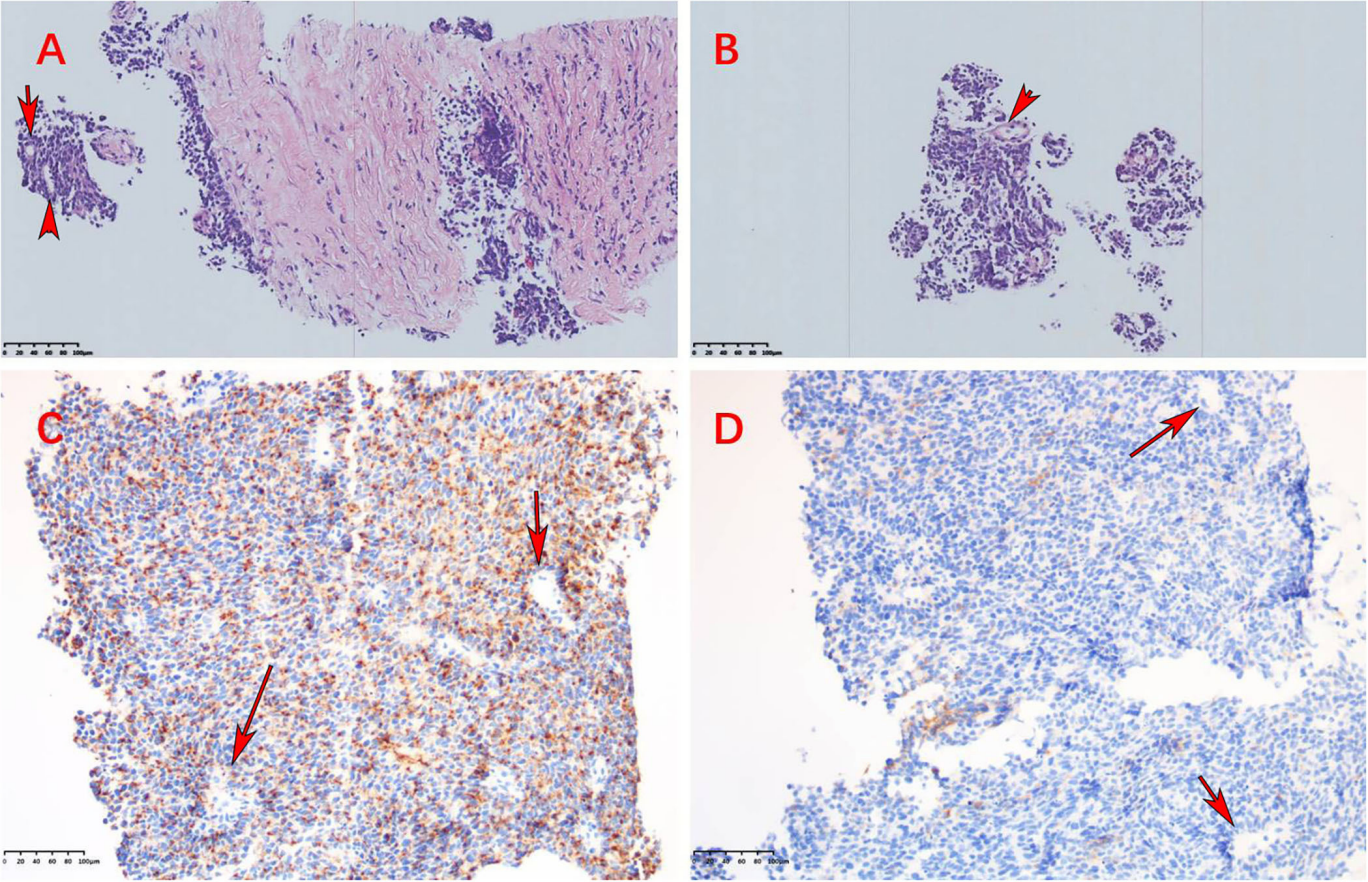
**(A,B)** High-power view of lung biopsy histopathology showed irregular multilayered rosettes composed of small round blue cells with atypia, respectively (HE, ×200). **(C,D)** Immunohistochemical staining was positive for EMA and Syn, respectively (×100) (these red arrows indicate multiple rosettes).

Postoperative radiotherapy was performed from January 2016 to March 2016, with a dose of 36 Gy in 24 fractions for the whole brain and the spinal cord, and the dose of postoperative tumor bed was increased to 60 Gy. From April to August 2016, the patient received an EP regimen (etoposide + cisplatin) for six cycles of chemotherapy. No complications or side effects were observed, and the efficacy was found to be a complete response (CR). In February 2021, the patient developed paroxysmal cough and sputum. In April 2021, chest CT showed a huge space-occupying lesion in the lung with bronchial occlusion, about 12.9 × 12.5 cm in size (Figure 3A), with an unclear boundary with the mediastinum and a small amount of effusion in the left pleural cavity. Laboratory tests showed elevated NSE (87.7 ng/ml), while other tumor markers, such as AFP, CEA, and CA- 99, were normal. Routine biopsy pathology of left lung mass showed fibrous tissue and a small number of round cell tumors with crush injury, containing multiple layers of irregular rosettes (Figures 4A,B). Immunohistochemistry showed EMA (+) (Figure 4C), Syn (weak +) (Figure 4D), CK-P(+), CD56(+), CgA(–), CD99(–), Ki-67(70% +), TTF-1(–), GFAP(–), and Oligo-2(–). At this time, MRI showed no recurrence in the primary tumor bed area, and lung metastasis was considered in combination with lung puncture pathology and immunohistochemistry.

After a multi-disciplinary discussion, the radiation dose of 30 Gy in 10 fractions for lung metastasis was performed in May 2021. Chest CT reexamination in June 2021 (after radiotherapy) indicated that the lung metastasis was significantly smaller than before, about 2.6 × 4.2 cm in size (Figure 3B), with soft tissue shadow visible in the anterior mediastinum, accompanied by mediastinal gas and increased left pleural effusion. From June 2021 to October 2021, he received an EP regimen (etoposide + cisplatin) for six cycles of chemotherapy. In October 2021 (after chemotherapy), chest CT reexamination showed that the lung metastatic lesions continued to shrink, about 2.2 × 0.9 cm in size (Figure 3C), and there was a large pleural effusion on the left side. Laboratory test results suggested that the NSE value decreased to normal (17.8 ng/ml), and the efficacy assessment was the partial response (PR). The most recent chest CT reexamination (February 2022) showed stable lung metastases, about 2.2 × 0.9 cm in size (Figure 3D).

## Follow-up

After comprehensive treatment, the patient was found to have pulmonary metastasis of ependymoblastoma and was followed up for 5 months. There was no local recurrence of the primary intracranial tumor, no significant change of the pulmonary metastasis, and a large amount of fluid is still accumulated in the left thoracic cavity. At present, the patient is generally in good condition, without neurological symptoms, respiratory symptoms, or any other discomfort; he is now living with the tumor.

## Discussion

Ependymoblastoma is a rare tumor of the central nervous system, often occurring in the supratentorial area in women and children, and its clinical manifestations are mainly elevated intracranial pressure, epileptic seizures, hemiplegia, cerebellar ataxia, and cranial nerve palsy (1). Ependymoblastoma is a PNET according to the 2007 CNS Tumor Classification Guidelines. In the 2016 update of CNS tumor classification guidelines, ependymoblastoma was redefined as an embryonic tumor with multilayer rosettes (ETMR), with amplification of the C19MC region on chromosome 19, rich in nerve cells and true rosettes (1, 2). Ependymoblastoma is an embryonic tumor derived from the neuroepithelium, with many rosettes and a small amount of proliferation of vascular endothelium, which can be used to differentiate it from anaplastic ependymoma (7). Furthermore, ependymoblastoma imaging shows intratumoral hemorrhage and cysts, with a poor prognosis (2, 8). However, it is easy to be misdiagnosed only by imaging examinations. A patient with ependymoblastoma was misdiagnosed as pilocytic astroglioma due to a lack of pathology and immunohistochemistry (9). Immunohistochemistry of ependymoblastoma is often focally positive for vimentin, s-100 protein, EMA, cytokeratin, synaptophysin, and CD99 (1, 3, 6).

In this case, the patient received GTR, which gave him a good outcome. The literature covered 71 cases of ependymoblastoma, 42 of which had complete clinical details, emphasizing the importance of GTR and chemotherapy, and indicating young age as a risk factor (10). Due-Tønnessen et al. (11) revealed that a 2-month-old patient with ependymoblastoma survived 12 years with good quality of life after GTR and postoperative chemotherapy and remains under follow-up. Due-Tønnessen et al. (11) showed that due to the younger age of the patient, the side effects of radiotherapy were uncontrollable, and radiotherapy could not be implemented, but the necessity of radiotherapy was also confirmed. Gerber et al. (12) revealed that 11 cases of patients with ependymoblastoma with a median age of 3.5 years, among which patients who underwent GTR combined with radiotherapy and chemotherapy had a long survival, with the longest DFS being 12.7 years. Multi-mode treatment was recommended to achieve sustained remission. Jaramillo et al. (13) discussed seven patients with ETMR who received proton therapy; this proved the importance of a maximum safe surgical range and radiotherapy and chemotherapy for ETMR. The tumor stages of the patients in their study were all time-limited M0, and they received tumor or tumor bed radiotherapy with a dose of 50.4 Gy/54 Gy. Among them, six patients received surgical treatment and cranial spinal cord irradiation at a dose of 36 Gy. All patients received systemic chemotherapy, including vp-16, VCR, VLB, MTX, DDP, IFO, and other treatments. The median overall survival (OS) was 16 months (range 8–64 months). Some studies have shown the efficacy of ETMR therapy, adding doxorubicin, actinomycin D, and other drugs to improve the patient's outcome (3). Based on this, an international consensus was developed. The efficacy of personalized molecular targeted therapy for ETMR is also being explored (14). Proton radiotherapy has a far more adjustable dose distribution than high-energy X-ray radiotherapy, and its Bragg peak can realize the rapid dose drop behind the tumor, which can help protect the organs at risk. Proton radiotherapy may play a role in reducing adverse reactions in the treatment of children's tumors (13). From the previous literature, we know that GTR combined with chemoradiotherapy is beneficial to the survival of patients. In this case, the tumor burden in the patient's lungs was large, but it was partially relieved by treatment. The patient is currently in stable condition.

## Conclusion

This case describes an atypical adolescent supratentorial ependymoblastoma with pulmonary metastasis. Our case underlines the importance of a regular follow-up, including a chest CT scan or PET. This case indicates that the lung metastases of ependymoblastoma are relatively sensitive to radiation, but the ideal radiotherapy dose for lung metastases needs to be further explored.

## Data availability statement

The original contributions presented in the study are included in the article/supplementary material, further inquiries can be directed to the corresponding author.

## Ethics statement

The patient has provided informed consent for publication of the case and any accompanying figures. Ethics Committee approval was received for this case report from the Ethics Committee of Jinhua Municipal Central Hospital.

## Author contributions

XinX was responsible for the writing of the article. AL and XiaX provided pathological and immunohistochemical data. QG and SZ were responsible for case data collection and image analysis. WC performed quality control of case data. SD was responsible for the revision of the article and finalized the first draft.

## Funding

This study was funded by the Medical and Health Science and Technology Project of Zhejiang Province (No. 2020KY1002, File No. 201947).

## Conflict of interest

The authors declare that the research was conducted in the absence of any commercial or financial relationships that could be construed as a potential conflict of interest.

## Publisher's note

All claims expressed in this article are solely those of the authors and do not necessarily represent those of their affiliated organizations, or those of the publisher, the editors and the reviewers. Any product that may be evaluated in this article, or claim that may be made by its manufacturer, is not guaranteed or endorsed by the publisher.
